# Suicidality, care access, and knowledge deficits lead priorities on blast overpressure/mTBI in an Australian special operations forces survey and their implications for evolving U.S. Warfighter brain health initiatives

**DOI:** 10.3389/fpubh.2026.1815984

**Published:** 2026-09-04

**Authors:** Rebecca A. Ivory, Paul Scanlan, Ricky Ditzel Jr, James S. Meabon

**Affiliations:** 1 School of Nursing, Emory University, Atlanta, GA, United States; 2 School of Nursing, University of Delaware, Newark, DE, United States; 3 Vigil Australia, Perth, WA, Australia; 4 Rush University Medical College, Chicago, IL, United States; 5 VA Northwest Mental Illness Research Education and Clinical Center (MIRECC), VA Puget Sound Health Care System, Seattle, WA, United States; 6 Department of Psychiatry, School of Medicine, University of Washington, Seattle, WA, United States

**Keywords:** clinical care access, community survey, depression, exposure history, health education, mild traumatic brain injury, military public health, suicidality

## Abstract

**Introduction:**

Blast overpressure (BOP) and mild traumatic brain injury (mTBI) create persistent health, readiness, and care-navigation challenges in Special Operations Forces (SOF). We conducted a stakeholder-informed needs assessment to identify priorities within an Australian SOF-affiliated community and to consider, without assuming direct generalizability, how those priorities may inform allied brain-health implementation.

**Methods:**

An anonymous web-based convenience survey was distributed through established Australian SOF-affiliated networks (187 submitted records). The purpose-built instrument collected demographic, service, BOP-exposure, symptom-category, community-issue, audience, tool, and educational-format information. Analyses were descriptive; ranked items were summarized with weighted priority scores.

**Results:**

Among respondents to the nationality item, 96.2% were Australian; 97.3% of all respondents were male, and 56.6% of service-status respondents were Veterans with SOF backgrounds. Median self-reported career BOP exposure was 13.5 years. Perceived medical-knowledge deficits, difficulty obtaining timely information, and limited access to specialized care were leading community issues. Suicidality, depressed mood and isolation, chronic brain changes, and attention/executive dysfunction received the highest weighted symptom-priority scores. Exposure-history estimation, event tracking, and self-administered baseline or symptom-monitoring tools were prioritized. Brief videos, linked reading materials, and testimonials were favored formats.

**Discussion:**

These findings provide directional, Australian-specific guidance for co-design and implementation testing under Recommendation 61 of the Royal Commission into Defence and Veteran Suicide. Their convergence with a separately conducted U.S. SOF-affiliated survey identifies candidate allied implementation themes, but the Australian sample should not be treated as representative of Australian SOF as a whole or directly generalizable to U.S. military populations.

## Introduction

Repetitive exposure to blast overpressure (BOP) and other neurotraumatic forces is common in tactical occupations (1–3). Mild traumatic brain injury (mTBI) refers to a traumatically induced alteration in brain function at the mild end of the TBI spectrum. Many occupational BOP events occur without a clinically recognized concussion or more severe TBI, yet cumulative exposure across years of service may contribute to cognitive, affective, vestibular, sensory, and other health concerns (2, 4–7). Australian and U. S. Defence and Veteran health systems increasingly treat brain health as both a readiness and a lifelong-care issue, while recognizing that implementation remains constrained by heterogeneous exposures, uncertain occupational thresholds, variable symptom trajectories, and uneven access to specialized expertise (1).

In Australia, the Department of Defence and Department of Veterans’ Affairs (DVA) are advancing an integrated brain-health approach spanning enlistment through post-service life, with explicit attention to blast exposure and neurocognitive outcomes (8). Recommendation 61 of the Royal Commission into Defence and Veteran Suicide specifically called for Defence and DVA to establish a brain-injury program that would improve understanding and mitigation of repetitive low-level blast effects, assessment and treatment of neurocognitive problems in serving and ex-serving members, and monitoring and recording of BOP exposure (9). Australian Defence and DVA subsequently established a Brain Injury Expert Advisory Panel and described ongoing work on exposure thresholds, monitoring, tracking, education, and evidence synthesis informed by Australian and international sources (10, 11). These activities make the Australian setting independently important and create an opportunity for cautious comparison with allied implementation efforts.

In the United States, the Department of Defense/War (DoD/W) Warfighter Brain Health Initiative (WBHI) links psychological and cognitive function, hazardous brain exposures, readiness, operational capability, and long-term outcomes for Service members and families (12, 13). Department-wide BOP requirements emphasize practical risk management, systematic exposure tracking, education concerning symptoms and performance effects, and phased baseline cognitive assessment (14, 15). U.S. Special Operations Command (USSOCOM) has also codified a career-lifecycle brain-health strategy integrating exposure monitoring, standardized neurocognitive assessment, and attention to cognitive and mental-health status (16). These policies provide a relevant comparison framework, but they do not make an Australian convenience sample representative of U.S. Personnel.

This policy context intersects with the public-health and readiness imperative of suicide prevention. The U.S. DoD recorded 523 Service member suicide deaths in calendar year 2023 (17). The 2023 VA National Veteran Suicide Prevention Annual Report documented 6,392 Veteran suicide deaths in 2021, averaging 17.5 per day (18). Peer-reviewed evidence also supports integrating suicide prevention with neurotrauma-focused care because concussion or mTBI history is associated with increased suicide risk in population-based analyses, and systematic reviews of military and Veteran cohorts link TBI exposure with psychiatric morbidity and suicidal behavior (19, 20). These associations do not establish that BOP caused suicidality in the present survey respondents. Rather, these associations explain why stakeholder prioritization of suicidality is clinically and operationally important.

Affected-community preferences are central to credible knowledge translation and implementation. The primary purpose of this stakeholder-informed quality-improvement (QI) needs assessment was to describe which BOP and mTBI-related community issues, symptom domains, dissemination audiences, practical tools, and educational formats were prioritized by an Australian Special Operations Forces (SOF)-affiliated convenience sample. A secondary purpose was to identify candidate themes relevant to allied brain-health implementation by comparing the direction of these Australian priorities with an independently conducted U.S. SOF-affiliated stakeholder survey using the same core framework (21). The Australian project was not designed to estimate population prevalence, test causal hypotheses, replace direct U.S. sampling, or support unqualified extrapolation to U.S. military populations.

## Methods

### Study framework and design

This project was undertaken as a stakeholder-engaged community-feedback and QI needs assessment intended to guide practical improvements in BOP and mTBI-related education, symptom monitoring, exposure documentation, and care navigation. It was not designed as a hypothesis-testing epidemiologic study. A conventional scientific structure is used for readability, but interpretation should remain aligned with community-engagement and QI principles (21–23).

### Participants, recruitment, and survey completion

Eligible participants were adults who self-identified as members of, or stakeholders in, the Australian SOF community, including current or former operators or enablers, other Service members or Veterans, spouses or partners, caregivers, and civilians engaged with the community. Vigil Australia, an independent, nonprofit and unfunded advocacy initiative, hosted the anonymous web-based survey over a continuous three-month period. Distribution was electronic and used a shareable survey link circulated through established SOF-affiliated online networks and community channels. Recipients could further share the link with adult stakeholders known to them. The survey was not administered by telephone or paper mail, and no individually targeted reminders were sent. Recruitment was convenience-based and network-mediated rather than probability-based. The analytic cohort comprised 187 submitted survey records. Items were optional, so submitted records could contain item-level missingness. All available responses were analyzed question by question.

### Survey development and measures

The Australian BRIDGES survey adapted the purpose-built core instrument used in a parallel U. S. SOF-affiliated QI activity (21). That core instrument was developed over approximately 2 months through informal semi-structured background interviews, ongoing stakeholder consultation, and repeated draft review by approximately 22 people with relevant lived or professional expertise, including current or former SOF Service members, personnel with Special Operations medical experience, caregivers, and an interdisciplinary group of clinician-scientists and allied health professionals (21). Review focused on content relevance, clarity, terminology, reading level, formatting, and practical actionability. The Australian adaptation retained the shared priority domains and added Australian-specific demographic, age, service, occupational, and exposure questions. Respondents reported demographic and service characteristics; years of service and BOP exposure using pre-specified four-year response categories (for example, 0–3, 4–7, and 8–11 years); exposure to blast-producing weapons and non-blast forces; and the presence of common symptom complaints. The symptom checklist assessed presence only and did not determine diagnosis, cause, duration, timing, functional impairment, or severity. Priority items addressed community issues, symptom domains, publication audiences, practical tools, preferred educational formats, and which product category should be developed first. The community-issues item used a predefined list without an item-specific “other” option. Occupational specialty was provided as a free-text query and coded to Australian military Employment Category Numbers (ECN). A general free-text comment field was available at the end of the survey but was not formally coded for this report. A final question asked respondents how the research team should consider further community engagement actions and is beyond the scope of the current report. The instrument was designed for pragmatic prioritization, not psychometric measurement. Formal test–retest reliability, internal-consistency, construct-validity, or criterion-validity studies were not performed, and the results are not presented as scores from a validated scale.

### Analysis

Categorical responses were summarized as counts and percentages using item-specific denominators. For years of service and BOP exposure, approximate means and standard deviations were estimated from the midpoints of the prespecified four-year response categories; ranges reflect the minimum and maximum category bounds observed. For ranking items, weighted priority scores used a 4–1 scheme (rank 1 = 4 points, rank 2 = 3, rank 3 = 2, and rank 4 = 1). Target-audience distributions were summarized descriptively across five rank positions. Because only aggregate counts were available for the present analysis, respondent-level linkage across questions was not possible; subgroup analyses by age, stakeholder role, service duration, medical background, or other characteristics could not be performed. Aggregate rank counts were analyzed as supplied, and any tied or repeated rank assignments could not be resolved without respondent-level records.

### Use of generative artificial intelligence

Following initial peer review, OpenAI ChatGPT, a generative artificial-intelligence large language model, was used to assist with editorial restructuring, language refinement, internal-consistency checking, and formatting of the revised manuscript. It was not used to collect, generate, impute, or analyze survey data; calculate statistical results; determine outcomes; make autonomous scientific decisions or derive study conclusions. The authors reviewed and verified all revisions and remain responsible for the manuscript.

### Human participant protection

This activity involved an anonymous, voluntary survey intended to inform community-engagement and QI priorities. It involved no prospective assignment to an intervention and no evaluation of intervention effects on health-related biomedical or behavioral outcomes. Consistent with the institutional determination, the activity was classified as QI/not human subjects research under the Emory University “Special Operations Forces (SOF) Care Quality Improvement Survey” project (#4060). No directly identifying information was collected, and the available data were analyzed in aggregate.

## Results

A total of 187 survey records were submitted. Item-level denominators varied because no survey item was mandatory (for example, service status *N* = 182, nationality *N* = 186, years of service *N* = 178, and years of BOP exposure *N* = 178). Table 1 summarizes respondent characteristics. Among nationality respondents, 179/186 (96.2%) identified Australia and 7/186 (3.8%) reported another nationality. Non-Australian nationalities were aggregated to avoid potentially identifying small groups. Respondents were predominantly male (182/187, 97.3%). Among service-status respondents, 103/182 (56.6%) were Veterans with SOF backgrounds and 31/182 (17.0%) were Veterans without SOF backgrounds. Active-duty personnel, reservists, and civilians comprised 14.8, 4.4, and 7.1%, respectively. Occupational responses reflected combat-arms, SOF, and enabling roles. “Current or former operator” was the most frequently selected role (125/182; 68.7%). Roles as current or former enabler were endorsed by 22/182 (12.1%) of respondents, followed by other Service member or Veterans (24.2%), spouse/partners (2.2%), primary caregivers (2.2%), and children of an operator/enabler (0%). Among respondents reporting an identifiable trade, badged special operators (Commando and Special Air Service Regiments; SASR) accounted for 29 (24.8%). A further 11 respondents (9.4%) were Navy clearance divers reporting counter-terrorism or Tactical Assault Group (CD-CT/TAG) assignment, giving 40 respondents (34.2%) in badged operator or force-assigned counter-terrorism roles. Infantry, which is the principal feeder trade to Special Operations Command and sits outside the operator and enabler structure, accounted for 30.8%, consistent with the enabler, supporting and stakeholder population targets of the study. Four respondents explicitly identified combat-paramedic or medical-technician specialties, but medical training was not collected as a separate standardized variable. Officer-versus-enlisted status was likewise not captured in a standardized field. Age and other demographic information were collected, but the aggregate analytic export did not permit respondent-linked subgroup analyses.

**Table 1:** Demographics of Australian special operations forces community survey respondents.

Characteristic	*n*	% or summary
Sex (item responses *n* = 187)		
Male	182	97.3
Female	5	2.7
Nationality (item responses *n* = 186)		
Australia	179	95.7%
Other non-Australian nationality (aggregated)	7	3.7%
Service status (item responses *n* = 182)		
Active Army (or active duty)	27	14.80%
Reserve Army (or reservist)	8	4.40%
Veteran, SOF background	103	56.60%
Veteran, non-SOF background	31	17.00%
Civilian	13	7.10%
Roles Endorsed (item responses *n* = 182; Select all that apply)		
Current or former operator	125	68.70%
Current or former enabler	22	12.10%
Other Service member or Veteran	44	24.20%
Spouse or partner	4	2.2%%
Primary caregiver	4	2.2%%
Child of operator or enabler	0	0%
Employment Category Number (ECN) (item responses *n* = 187)		
Other ECN or Veteran—unspecified service category	44	23.50%
343—Infantry	36	19.30%
079—Commando	24	12.80%
Current or former enabler—combat support personnel	22	11.80%
CD (CT)—CD (CT/TAG)	11	5.90%
CD—Navy	9	4.80%
401—Ammunition tech (RAAOC)	7	3.70%
353—SASR	5	2.70%
662—Signaller (RASIGS)	5	2.70%
PTG—Police tactical group (non-military)	4	2.10%
096—Combat engineer (RAE)	3	1.60%
169—Combat paramedic (RAAMC)	3	1.60%
060—Armoured vehicle crew (RAAC)	2	1.10%
146—Fitter armament (RAEME)	2	1.10%
162—Gunner (RAA)	2	1.10%
Spouse or partner—family member, spouse, or partner	2	1.10%
Primary caregiver—primary caregiver for service member	2	1.10%
031—medical technician (RAAMC)	1	0.50%
109—driver (RACT)	1	0.50%
229—vehicle mechanic (RAEME)	1	0.50%
Unknown—RAAF	1	0.50%
Years of service (item responses *n* = 178)		
Years of service (years), mean ± SD [range]		18.4 ± 10.4 [0–51]
Years of service distribution (4-year bins)		
0–3	5	2.80%
4–7	13	7.30%
8–11	31	17.40%
12–15	38	21.30%
16–19	21	11.80%
20–23	24	13.50%
24–27	16	9.00%
28–31	7	3.90%
32–35	10	5.60%
36–39	3	1.70%
40–43	4	2.20%
44–47	4	2.20%
48–51	2	1.10%

Item-level denominators vary due to non-response. Years-of-service mean and SD were estimated from binned counts using interval midpoints; range reflects the observed bin endpoints. Percentages may not sum to 100% due to rounding.

**Figure 1:**
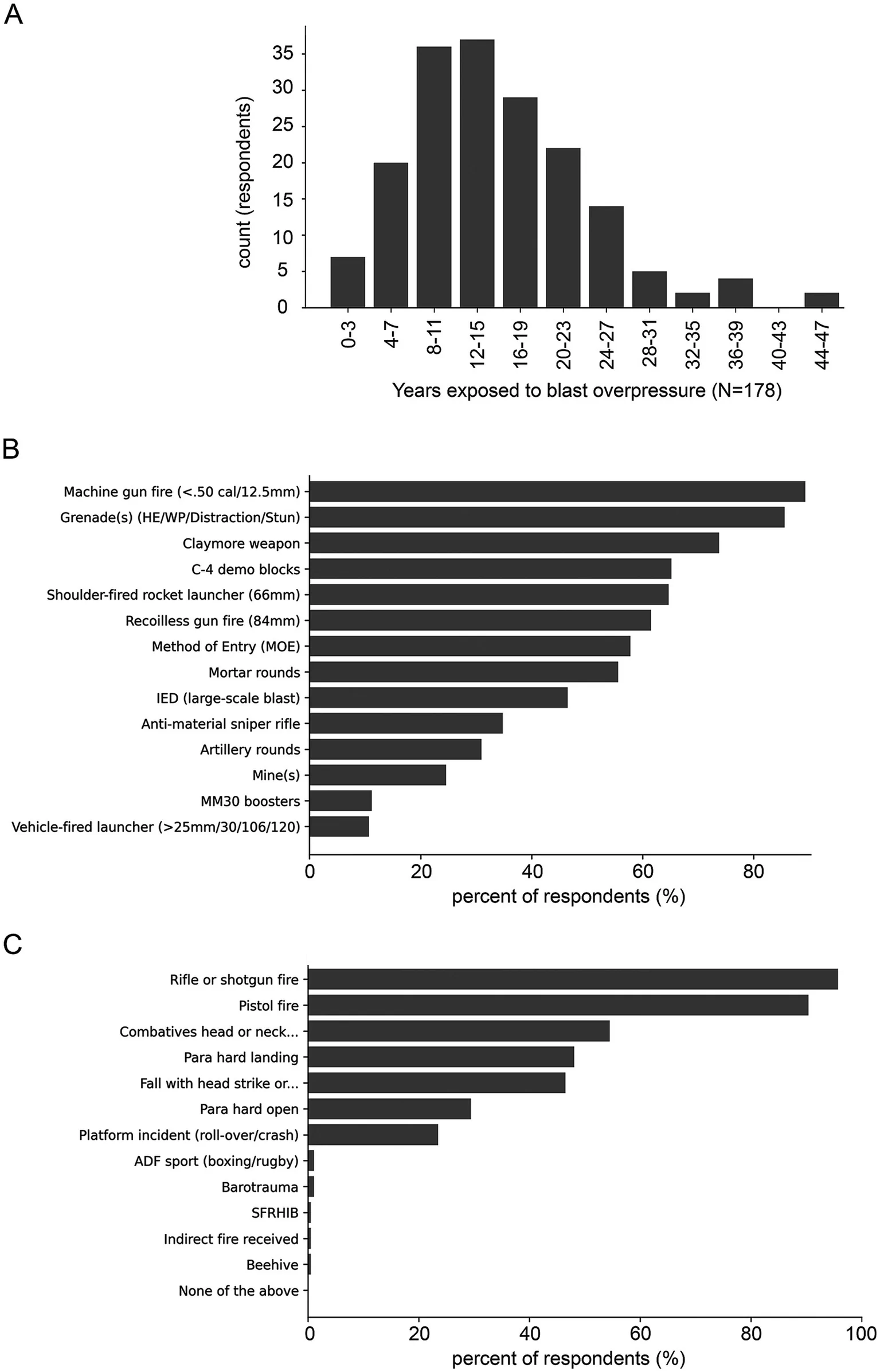
Self-reported blast overpressure (BOP) and non-blast exposure categories. **(A)** Distribution of respondents across prespecified categories of years exposed to BOP during service (*N* = 178). **(B)** Endorsements of blast-producing weapon systems (*N* = 187). **(C)** Endorsements of non-blast force exposures (*N* = 187). Endorsements were not verified by records or dosimetry.

Among respondents providing service-duration data (*N* = 178), approximate mean service duration was 18.4 ± 10.4 years (range, 0–51 years). Years of BOP exposure were concentrated between 8 and 23 years; the 8–11 and 12-15-year categories together included approximately 40% of respondents. Median self-reported BOP exposure was 13.5 years, and the approximate mean was 15.5 ± 8.4 years (Figure 1A). These exposure-duration summaries characterize the responding sample but do not objectively verify cumulative dose.

Respondents endorsed 1,332 blast-producing weapon-system exposures, corresponding to a mean of 7.1 endorsed systems per respondent. The most frequently endorsed sources were machine-gun fire (89.3%), grenades (85.6%), Claymore weapons (73.8%), demolition charges (65.2%), shoulder-fired rocket launchers (64.7%), and the recoilless Carl Gustav 84 mm gun (61.5%), with additional endorsements involving breaching methods, mortars, and larger explosive hazards (Figure 1B). Respondents also made 733 non-blast force endorsements, or a mean of 3.9 per respondent, including rifle or shotgun fire (95.7%), pistol fire (90.4%), combatives-related head or neck impacts (54.5%), hard parachute landings (48.1%), and falls with head strike (46.5%) (Figure 1C). These were self-reported exposure categories and were not verified using records or dosimetry.

**Figure 2:**
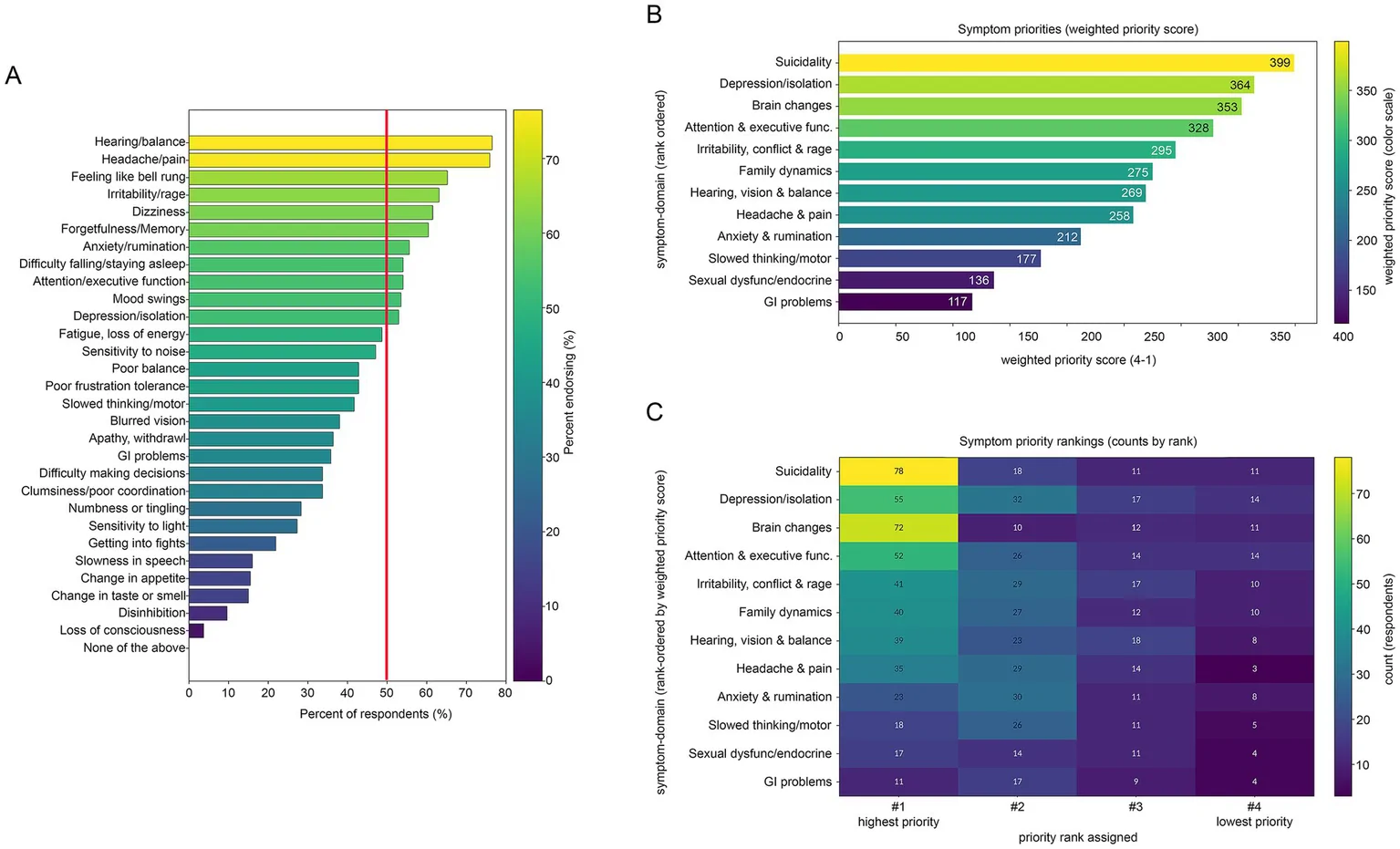
Symptom-category endorsements and symptom-domain priorities. **(A)** Frequency of symptom-presence endorsements across respondents. Presence endorsements do not establish diagnosis, cause, duration, residual post-concussive syndrome, or severity. **(B)** Weighted symptom-domain priorities using 4–1 scoring. **(C)** Heatmap of aggregate rank counts across symptom domains for ranks 1–4. Panels **(A–C)**, *N* = 187.

The symptom-presence checklist yielded 2,264 endorsements, corresponding to a mean of 12.1 endorsed symptom categories per respondent. The most frequently endorsed categories included hearing or balance difficulties (76.5%), headaches or pain (75.9%), feeling as though one’s “bell was rung” (65.2%), irritability or easy annoyance (63.1%), dizziness (61.5%), forgetfulness or memory problems (60.4%), anxiety or rumination (55.6%), sleep-initiation or maintenance problems (54.0%), attention/executive function difficulty (54.0%), mood swings (53.5%), and depressed mood or isolation (52.9%) (Figure 2A). Loss of consciousness was endorsed by 3.7% of respondents, against 65.2% endorsing feeling like one’s ‘bell was rung’, a contrast consistent with a predominantly sub-concussive exposure profile. These endorsements do not establish clinical diagnosis, residual post-concussive syndrome, symptom severity, or attribution to BOP or TBI.

When respondents prioritized symptom domains for future education or research, suicidality received the highest weighted priority score (399), followed by depressed mood and isolation (364), chronic brain changes (353), and attention, concentration, time-management, and decision-making changes (328). Irritability, conflict and rage (295) and family or intimate-partner dynamics (275) also ranked highly, whereas gastrointestinal problems and sexual-function or endocrine changes received lower aggregate priority scores (Figures 2B,C; Supplemental Table S1). These rankings concern publication and implementation priorities and should not be interpreted as prevalence or severity estimates.

**Figure 3:**
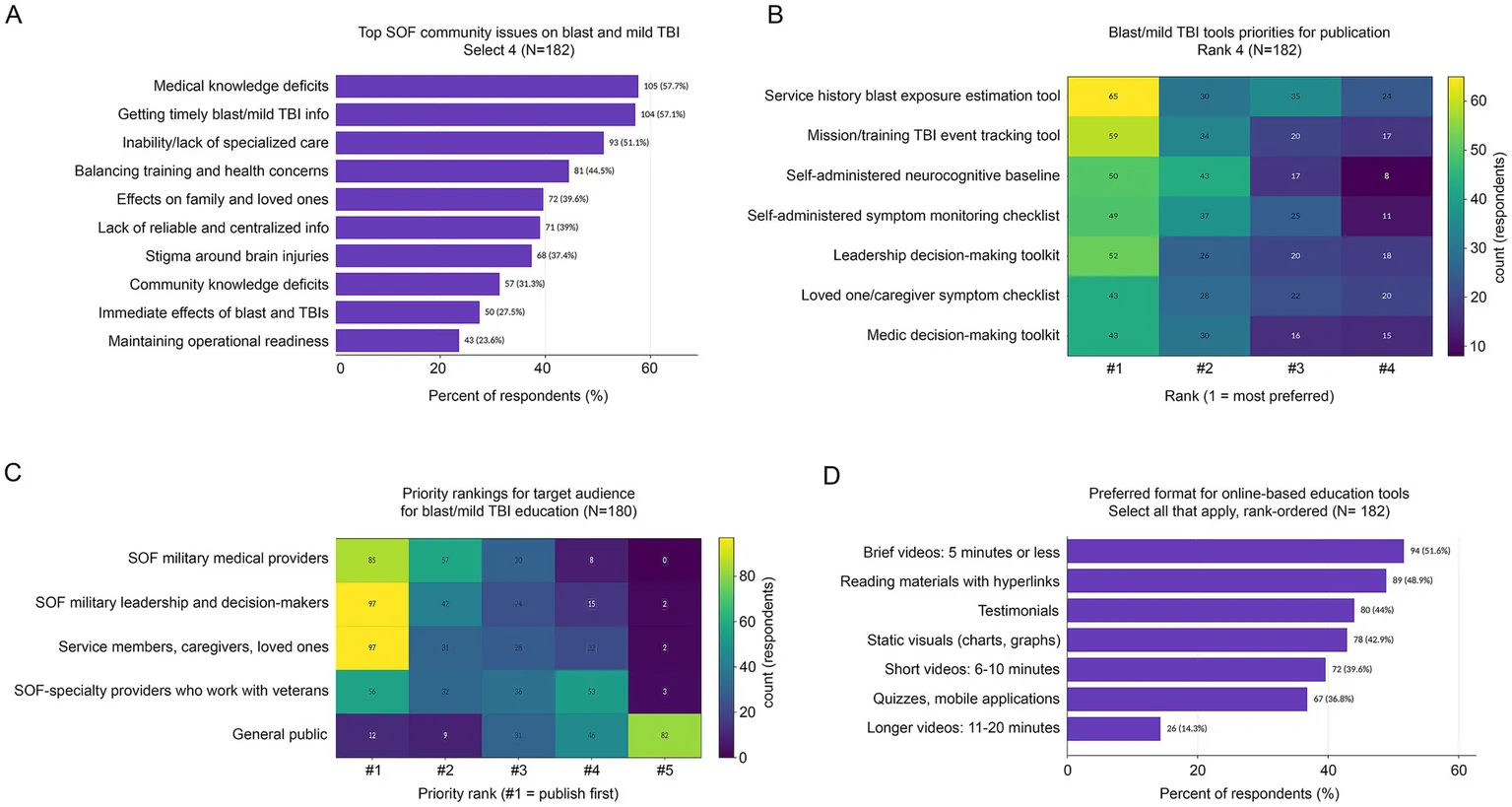
Perceived community issues, tool priorities, target audiences, and educational formats. **(A)** SOF-community issues related to BOP and mTBI (select four; *N* = 182). **(B)** Tool priorities (ranks 1–4; *N* = 182). **(C)** Publication-audience priorities (five rank positions; *N* = 180). **(D)** Preferred online educational formats (select all that apply; *N* = 182). Rank distributions are descriptive aggregate counts; respondent-level ties or repeated assignments could not be resolved.

For perceived community issues related to BOP and mTBI (select four; *N* = 182), the most frequently selected items were perceived medical-knowledge deficits (57.7%), difficulty obtaining timely BOP or mTBI information (57.1%), inability or lack of access to specialized care (51.1%), balancing training requirements with health concerns (44.5%), and effects on family and loved ones (39.6%) (Figure 3A). The survey measured respondent perceptions of these gaps; it did not audit the medical literature, clinician competence, or health-system performance.

For practical-tool priorities (*N* = 182), the service-history BOP-exposure estimation tool had the highest weighted score (444), followed by a mission or training TBI-event tracking tool (395), a self-administered neurocognitive baseline (371), and a self-administered symptom-monitoring checklist (368). Leadership, caregiver, and medic decision-support tools also received support but had lower aggregate scores (Figure 3B).

For publication audiences (*N* = 180), the highest-priority rank positions were concentrated among SOF military leadership and decision-makers, Service members and their caregivers or loved ones, and SOF military medical providers. The general public was most often placed in the lowest-priority position (Figure 3C). Because respondent-level rankings were unavailable and repeated or tied assignments could not be resolved, these patterns are presented descriptively rather than as a strict rank-order comparison.

For online educational formats (select all that apply; *N* = 182), brief videos of 5 min or less were most frequently selected (51.6%), followed by linked reading materials (48.9%) and personal testimonials (44.0%). Longer videos of 11–20 min were selected less frequently (14.3%) (Figure 3D).

When asked which product category should be published first, respondents selected symptom-focused (41.8%) and education-focused products (41.2%) at similar rates, whereas tools were selected by 17.0%. Collectively, these results describe the priorities of the responding convenience sample and should not be considered representative estimates for all Australian SOF personnel, Veteran, family, or caregiver populations.

## Discussion

This stakeholder-informed QI needs assessment identified directional priorities for BOP and mTBI-related process improvement within an Australian SOF-affiliated convenience sample. Respondents emphasized perceived gaps in medical knowledge, timely and reliable information, and access to specialized care, while also prioritizing exposure documentation, symptom monitoring, mental-health risk, and cognitive or neurological concerns. The study was designed to support practical prioritization and co-design, not to estimate population prevalence, establish causal effects of BOP, or mandate policy.

These findings have particular relevance to the Australian policy and service context. Recommendation 61 of the Royal Commission into Defence and Veteran Suicide, which remains agreed in principle and under detailed consideration by the Royal Commission into Defence and Veteran Suicide Taskforce, calls for improved approaches to monitoring and recording blast exposure. In parallel, Defence and DVA have established a Brain Injury Expert Advisory Panel that is considering exposure thresholds, monitoring and recording practices, and education. The two highest-ranked tools in the present survey—a service-history exposure estimation tool and a mission- and training-event tracking tool—align closely with these priorities and provide community-informed evidence that may help guide development of practical monitoring and recording approaches. Access to clinicians with specialized expertise, identified as a need by 51.1% of respondents, also highlights an issue that spans both Defence health services and DVA clinical pathways. Respondents further emphasized the broader social consequences of blast and mTBI: family and intimate-partner dynamics received a weighted priority score of 275, and 39.6% identified effects on family and loved ones as an important concern. These findings support development of family-facing education and resources alongside products directed toward serving members and veterans. Finally, DVA has commissioned the University of New South Wales to undertake a rolling review of international evidence concerning mTBI and low-level blast exposure. The priorities identified here can complement that evidence-synthesis effort by indicating which aspects of the evolving scientific literature the Australian SOF community most wants translated into accessible information, clinical guidance, and practical tools.

The knowledge and care-access findings are important but require disciplined interpretation. Respondents were asked whether these were community concerns; they were not asked to demonstrate deficiencies in the scientific literature or to evaluate clinician competence. Only four respondents explicitly listed combat-paramedic or medical-technician specialties, and medical training was not captured as a standardized characteristic. The results therefore support engaging Australian clinicians, medics, operators, leaders, Veterans, and families in co-designing educational and navigation resources, followed by prospective testing of whether those resources improve knowledge, trust, referral, or care access.

Suicidality and depressed mood or isolation received the highest symptom-priority scores. This finding does not show that suicidality was caused by BOP, nor does it measure current suicide risk or symptom severity. It shows that many respondents considered suicide-related and depressive outcomes especially urgent topics for future education and decision support. That priority is consistent with Australian and U.S. suicide-prevention imperatives and with literature linking TBI history to psychiatric morbidity and suicidal behavior (17–20). A reasonable implementation response is to pair crisis-oriented content and clear escalation pathways with earlier indicators that respondents also prioritized, including cognitive change, irritability, sleep difficulty, pain, social withdrawal, and family strain. Any such product should be developed with suicide-prevention specialists and evaluated prospectively rather than assumed effective from preference data alone.

High priority for chronic brain changes and attention or executive dysfunction indicates concern about both immediate safety and long term function. Tool preferences similarly favored exposure-history estimation, event tracking, neurocognitive baselines, and symptom monitoring. These priorities are directionally consistent with Australian Recommendation 61 and with U.S. BOP policies emphasizing exposure documentation, education, and baseline assessment (9, 14–16). The survey does not validate a specific instrument or threshold. Instead, it provides candidate product requirements indicating that tools should be usable in high-tempo settings, connect exposure and symptom information to clear next steps, protect privacy, and interface with established clinical pathways rather than operate as stand-alone diagnostic devices (24).

Respondents also prioritized internal military audiences, medical providers, Service members, and families over broad public dissemination, and they favored brief videos, linked reading materials, and testimonials. The survey did not ask why these audiences or formats were selected, so explanations concerning trust, gateway roles, attention burden, or operational tempo remain provisional. Nonetheless, these preferences justify co-designing short, role-specific prototypes for clinicians or medics, leaders, operators, Veterans, and families and then testing their acceptability, comprehension, reach, and implementation impact.

The relevance to U.S. Warfighter Brain Health efforts is comparative rather than directly generalizable. The Australian survey was not intended to substitute for direct U.S. sampling; a separate U.S. SOF-affiliated stakeholder survey using the same core framework has now been published (21). Across the two convenience samples, several themes converged highlighting shared perceived medical-knowledge and information-access gaps, difficulty reaching specialized care, high priority for cognitive and affective concerns, interest in exposure or event tracking and self-monitoring tools, preference for internal clinical or operational audiences, and preference for brief or experience-grounded educational formats (21). Identifying such gaps provides development opportunities that may leverage potentially shared cost and risk mitigation. Critically, the findings provide allied-military validation of a core WBH Initiative premise, arguing that Warfighter-facing priorities (including stigma, access, and practical guidance) are not peripheral, but rather critical implementation constraints that determine whether brain health policy translates into reduced morbidity and improved Warfighter readiness. Important differences were also evident. Specifically, suicidality and exposure-history estimation were especially prominent in the Australian weighted priorities, whereas the U.S. sample gave the highest aggregate scores to attention or decision-making changes and self-administered neurocognitive baselines (21). This cross-national convergence and divergence provide hypotheses and implementation questions for allied programs; they do not erase cultural, organizational, health-system, or sampling differences between Australia and the United States.

### Strengths and limitations

Strengths include a comparatively large SOF-affiliated allied sample for a pragmatic needs assessment, substantial reported service and BOP-exposure experience, assessment of multiple exposure sources and symptom categories, and direct alignment with an active Australian policy window. Several limitations constrain interpretation. Recruitment was convenience-based, voluntary, and network-mediated, creating self-selection and unknown response-rate bias. The sample was predominantly male and weighted toward Veterans, with small numbers of spouses, caregivers, and civilians. Exposure and symptom-presence data were self-reported and were not verified clinically or with dosimetry; symptom severity, duration, temporal relation to exposure, functional impairment, and clinical diagnoses were not measured. The purpose-built instrument underwent stakeholder and expert refinement but not formal psychometric reliability or validity testing. Available aggregate data prevented respondent-linked analyses across questions and subgroup comparisons by stakeholder type, age, service duration, medical background, officer or enlisted status, exposure history, or symptom burden. Non-Australian nationalities were aggregated, and rank ties or repeated assignments could not be resolved. Finally, Australian and U.S. military cultures, policies, care systems, and suicide-related contexts may differ. The findings should therefore be interpreted as descriptive stakeholder input from a limited Australian SOF-affiliated sample.

### Public health implications

For Australian Defence, DVA, and SOF-community stakeholders, the present results identify candidate priorities for iterative developments that improve clinician and medic knowledge, make reliable information easier to access, strengthen pathways to specialized care, incorporate suicide-prevention and depression content, and develop practical exposure and symptom-documentation tools. For U.S. WBHI and USSOCOM programs, the findings are most useful as an allied comparison that can challenge, corroborate, or refine locally collected U.S. stakeholder data rather than as evidence of U.S. preferences (12, 16, 21). The next step is not broad deployment based on a single survey. It is structured co-design with underrepresented stakeholder groups, confirmation using probability-informed or broader recruitment where feasible, respondent-level subgroup analysis, and prospective evaluation of whether specific educational products, tracking tools, and care-navigation workflows are feasible, acceptable, equitable, and associated with measurable improvements in knowledge, documentation, referral, care engagement, readiness, or health outcomes.

## Data Availability

The original contributions presented in the study are included in the article/Supplementary material, further inquiries can be directed to the corresponding author.
