# Supplementary material for: Suicidality, care access, and knowledge deficits lead priorities on blast overpressure/mTBI in an Australian special operations forces survey and their implications for evolving U.S. Warfighter brain health initiatives
Source: Front Public Health. 2026 Sep 4;14:1815984. doi: 10.3389/fpubh.2026.1815984 (PMC13598961; doi:10.3389/fpubh.2026.1815984)
Supplement: Supplementary file 1 [file Table_1.docx]

**Supplemental Table 1. Symptom-domain priority rankings (top-4 ranks reported) and weighted priority scores**

| **Symptom domain** | **#1** | **#2** | **#3** | **#4** | **Total selections** | **Weighted priority score (4–1)** |
| --- | --- | --- | --- | --- | --- | --- |
| Suicidal thoughts & behaviors | 78 | 18 | 11 | 11 | 118 | 399 |
| Depressed mood & isolation | 55 | 32 | 17 | 14 | 118 | 364 |
| Chronic brain changes | 72 | 10 | 12 | 11 | 105 | 353 |
| Attention/concentration, time management, & decision making changes | 52 | 26 | 14 | 14 | 106 | 328 |
| Irritability, conflict & rage | 41 | 29 | 17 | 10 | 97 | 295 |
| Family & intimate partner dynamics | 40 | 27 | 12 | 10 | 89 | 275 |
| Hearing, vision & balance changes | 39 | 23 | 18 | 8 | 88 | 269 |
| Headaches & chronic pain | 35 | 29 | 14 | 3 | 81 | 258 |
| Anxiety & rumination | 23 | 30 | 11 | 8 | 72 | 212 |
| Slowed thoughts & movements | 18 | 26 | 11 | 5 | 60 | 177 |
| Sexual dysfunction & endocrine changes | 17 | 14 | 11 | 4 | 46 | 136 |
| Gastrointestinal problems | 11 | 17 | 9 | 4 | 41 | 117 |

Notes: Weighted priority score assigns 4 points to rank #1, 3 points to rank #2, 2 points to rank #3, and 1 point to rank #4.
